# Computational Analysis of Bone Remodeling in the Proximal Tibia Under Electrical Stimulation Considering the Piezoelectric Properties

**DOI:** 10.3389/fbioe.2021.705199

**Published:** 2021-09-08

**Authors:** Yogesh Deepak Bansod, Maeruan Kebbach, Daniel Kluess, Rainer Bader, Ursula van Rienen

**Affiliations:** ^1^ Institute of General Electrical Engineering, University of Rostock, Rostock, Germany; ^2^ Department of Orthopaedics, Rostock University Medical Center, Rostock, Germany; ^3^ Department Life, Light and Matter, University of Rostock, Rostock, Germany; ^4^ Department Ageing of Individuals and Society, University of Rostock, Rostock, Germany

**Keywords:** bone remodeling, piezoelectricity, therapeutic electrical stimulation, finite element analysis, open-source, human tibia, hounsfield units (HU), bone mineral density (BMD)

## Abstract

The piezoelectricity of bone is known to play a crucial role in bone adaptation and remodeling. The application of an external stimulus such as mechanical strain or electric field has the potential to enhance bone formation and implant osseointegration. Therefore, in the present study, the objective is to investigate bone remodeling under electromechanical stimulation as a step towards establishing therapeutic strategies. For the first time, piezoelectric bone remodeling in the human proximal tibia under electro-mechanical loads was analyzed using the finite element method in an open-source framework. The predicted bone density distributions were qualitatively and quantitatively assessed by comparing with the computed tomography (CT) scan and the bone mineral density (BMD) calculated from the CT, respectively. The effect of model parameters such as uniform initial bone density and reference stimulus on the final density distribution was investigated. Results of the parametric study showed that for different values of initial bone density the model predicted similar but not identical final density distribution. It was also shown that higher reference stimulus value yielded lower average bone density at the final time. The present study demonstrates an increase in bone density as a result of electrical stimulation. Thus, to minimize bone loss, for example, due to physical impairment or osteoporosis, mechanical loads during daily physical activities could be partially replaced by therapeutic electrical stimulation.

## Introduction

The human skeletal system consists of bones and joints, which maintains the structural integrity of the body, provides sites for muscle attachment, and facilitates body movements (Cowin, 2001). Bone adapts its structure in response to changes in its mechanical loading environment and this adaptation process is known as bone remodeling (Robling and Turner, 2009). This process has a significant impact on the individual’s health and thus bone remodeling study is of prime importance. Additionally, piezoelectricity plays a vital role in bone adaptation and remodeling processes (Mohammadkhah et al., 2019). Therefore, in order to achieve better understanding of mechanical and electrical interactions that occur during these processes, computational analysis of piezoelectric bone remodeling is of great interest in musculoskeletal biomechanics.

In the literature, many mathematical models of bone remodeling are based on the qualitative observations of Wolff (1893), and these models have been implemented using the finite element method to simulate the bone response to mechanical loading (Weinans et al., 1992; Fernández et al., 2010; Fernández et al., 2012a; Garzón-Alvarado et al., 2012; Mauck et al., 2016; Garijo et al., 2017; Quilez et al., 2017) and have been reviewed in (Gerhard et al., 2009). The bone remodeling algorithms proposed in the past are based on strain (Wang et al., 2016), stress (Gong et al., 2013), strain energy density (SED) (Weinans et al., 1992; Fernández et al., 2010; Bouguecha et al., 2011), deformation (Papathanasopoulou et al., 2002), and mechanical damage (Garcia-Aznar et al., 2005; Hambli et al., 2011) or a combination of these, which elucidate many aspects of bone adaptation. The rate of change in bone density can be linear or nonlinear with respect to the applied mechanical load. Moreover, the increment and decrement in this rate can be similar or different (Su et al., 2019). Although bone remodeling has multiple aspects, these are confined to study the response of bone to a particular loading type.

Piezoelectricity can explain the involvement of electrical signals and mechanical loads in the bone adaptation process. Yasuda (1954) was the first one to report the direct and converse piezoelectric properties of the dry bone and these findings have been confirmed by other investigators (Fukada and Yasuda, 1957; Johnson et al., 1980). They attributed the piezoelectric behavior of bone to matrix piezoelectricity, in which application of mechanical shear to collagen fibers generates electrical charges. It was found that the quasi-hexagonal symmetry of the collagen fiber is responsible for the shear piezoelectric effect in bone (Minary-Jolandan and Yu, 2009). Based on experimental observations (Pienkowski and Pollack, 1983; Hastings and Mahmud, 1988), the streaming potential causes piezoelectricity in wet bone and is the main source for strain-generated potentials (SGPs). Streaming potential arises from the fluid flow through charged surfaces. Mechanical deformation causes fluids to flow through the canaliculi and correspondingly ions flow is generated against the oppositely charged walls, leading to a potential difference between two points along the stream (Mohammadkhah et al., 2019). Until now, the specific mechanisms for bone piezoelectricity remain unclear. However, there are few computational models of bone remodeling that take the piezoelectricity of bone into account (Qu et al., 2006; Fernández et al., 2012a; Garzón-Alvarado et al., 2012; Beheshtiha and Nackenhorst, 2015; Cerrolaza et al., 2017; Duarte et al., 2018) and have been summarized in recent review by Mohammadkhah et al. (2019).

Several studies have described the piezoelectric effects on bone biophysics. The linear piezoelectric theory for bone remodeling was presented by Gjelsvik (1973). It was observed in experimental studies that bone possesses electric and dielectric properties, which are frequency-dependent (Bassett and Becker, 1962; Bassett et al., 1964; Su et al., 2016) and these properties are important for the hypothesized feedback mechanism for the bone remodeling process and the therapeutic electrical stimulation for bone healing process (Ramtani, 2008). It has been demonstrated that the electromagnetic field affects the bone remodeling and healing process under the influence of mechanical and electrical loadings (Qu et al., 2006; Qu and Yu, 2011). Also, there are models that considered bone remodeling under the combined action of mechanical, electrical, and thermal loads (Qin, 2003; Qin et al., 2005). However, the effect of these stimuli on the remodeling process is still ambiguous. For example, an understanding of this could be useful for improving the implant design (Lian et al., 2014). Although there are many femoral bone remodeling studies, similar studies on tibial bone are very few (Perez et al., 2010; Robalo, 2011; Fang et al., 2013; González-Carbonell et al., 2015). Moreover, there is no investigation in the literature on bone remodeling in the human tibia considering its piezoelectric properties. Therefore, the aim of our present study is to investigate piezoelectric bone remodeling in the human tibia under electrical stimulation. This study contributes to a better understanding of tibial bone response to electromechanical loads that can aid in designing a protocol for therapeutic electrical stimulations to minimize bone loss, for example, due to physical impairment or osteoporosis.

## Material and Methods

### Mathematical Formulation of the Piezoelectric Bone Remodeling

**FIGURE 1:**
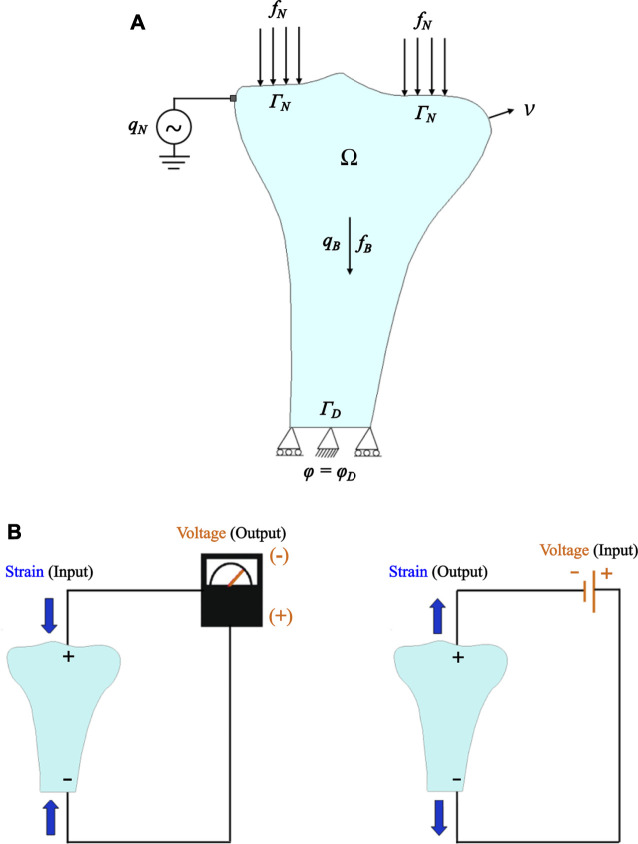
**(A)** The tibia bone domain and schematic representation of boundary conditions implemented for piezoelectric bone remodeling simulations under electrical stimulation. **(B)** Exemplary representation of the direct and converse piezoelectric effect in tibia bone.

Several bone remodeling algorithms have been proposed to compute the evolution of bone density under mechanical loadings; however, only a few of these algorithms have been considering bone piezoelectricity. Based on the piezoelectric strain-adaptive bone remodeling algorithm proposed by Fernández et al. (2012a), let Ω be an open-bounded domain (see Figure 1A), and Γ *=*  ∂Ω be its boundary. This boundary was considered to be Lipschitz continuous. It has been split into a Neumann boundary$\;\Gamma_{N}$ and a Dirichlet boundary $\Gamma_{D}$ *.* The density of volume forces acting in domain Ω is denoted by $f_{B}$, and the density of traction forces $f_{N}$ was applied on$\;\Gamma_{N}$. It has been assumed that the bone is fixed at $\Gamma_{D}$, i.e., here u = 0 holds for the displacement vector. Next, the density of volume electric charges present in domain Ω is represented by $q_{B}$ and the density of surface electric charges applied externally on$\;\Gamma_{N}$ is represented by $q_{N}$. Let φ be the electric potential. An electric potential $\varphi_{D}=0$ was applied to the fixed boundary (Fernández et al., 2012a). Let [0, T], T > 0 be the period of interest and ν(x) be the outward unit normal vector to Γ at a point x. Here, the same decomposition of the boundary has been used for imposing the boundary conditions for the mechanical displacement and the electric potential (Fernández et al., 2010). Note that the symbols in bold represent vectors, tensors, or matrices.

The linearized strain tensor ε(u) can be defined as

$$\varepsilon_{ij}\left(u\right)=\;\frac{1}{2}\;\left(\frac{\partial u_{i}}{\partial x_{j}}+\;\frac{\partial u_{j}}{\partial x_{i}}\right)\text{,}i,j=\;1\text{,}\ldots \;,d,$$ (1)

where u represents the displacement field and *d* is the order of symmetric matrices (3×3). Similar to Weinans et al. (1992), the bone was assumed to be isotropic and linear elastic. For the stress σ, the constitutive law can be written as follows:

$$\sigma =\sigma \left(u\right)=2\mu \left(\rho \right)\varepsilon \left(u\right)+\lambda \left(\rho \right)\text{Div}\left(u\right)\;ℐ-\alpha \left(\rho \right)\;{ℰ}^{*}\;E\left(\varphi \right)\;\text{in}\;\bar{\Omega }\times \left[0,T\right],$$ (2)

where μ(ρ) and λ(ρ) are Lamé coefficients and were considered to be dependent on the bone apparent density ρ, Div denotes the divergence operator, and *ℐ* represents the identity operator (Fernández et al., 2010). For the plane strain condition or the three-dimensional model, Lamé coefficients can be defined in terms of elastic modulus E(ρ) and Poisson’s ratio k(ρ) as follows:

$$\mu \left(\rho \right)=\;\frac{E\left(\rho \right)}{2\left(1+k\left(\rho \right)\right)}\;and\;\lambda \left(\rho \right)=\;\frac{k\left(\rho \right)E\left(\rho \right)}{1-k^{2}\left(\rho \right)}.$$ (3)

The Poisson’s ratio was considered to be not dependent on *ρ* (and thus, *k(ρ) = k*). The following equation was employed for elastic modulus depending on the apparent bone density:

$$E\left(\rho \right)=\;M\rho^{\gamma },$$ (4)

where M and γ are positive constitutive constants that characterize the behavior of bone (Weinans et al., 1992). Similar to the elastic modulus, a constitutive function α(ρ) was assumed to be dependent on the apparent bone density function and expressed as (Fernández et al., 2012a),

$$\alpha \left(\rho \right)=\;\rho^{\gamma }.$$ (5)

Further, **ℰ** ^**∗**^ denotes the transpose of the third-order piezoelectric tensor **ℰ** described below. As a conservative field, the stationary electric field **E** can be computed from the gradient of the electric potential φ (van Rienen, 2001):

E=−∇φ. (6)

The following first-order ordinary differential equation was employed to calculate the evolution of the apparent bone density function (Weinans et al., 1992),

$$\frac{d\rho }{dt}=B\left(\frac{U\;\left(\sigma \left(u\right),\;\;\varepsilon \left(u\right)\right)}{\rho }-\;S_{r}\right)\;\text{in}\;\;\Omega \times \left(0,\;T\right),$$ (7)

where the values of experimental constants B and $S_{r}$ are mentioned in Table 1. Further, the strain energy density (SED) as mechanical stimulus U(σ(u), ε(u)) can be given as:

$$U\left(\sigma \left(u\right),\varepsilon \left(u\right)\right)=\frac{1}{2}\;\sigma \left(u\right)\;:\varepsilon \left(u\right),$$ (8)

where “:” denotes the inner product and the apparent density has been considered to be restricted as follows:

$$\rho_{a}\;\leq \;\rho \;\leq \;\rho_{b},$$ (9)

where the minimum density $\rho_{a}$ and the maximum density $\rho_{b}$ correspond to the resorbed bone and the cortical bone, respectively. The constitutive law for the electric displacement D can be expressed as:

D= α(ρ) ℰ ε(u) + α(ρ)βE(φ), (10)

where β is the electric permittivity tensor (Batra and Yang, 1995). The constitutive equations for the stress σ (Eq. 2) and the electric displacement D (Eq. 10) govern the piezoelectric behavior of bone. When subjected to mechanical loading, bone generates an electric charge (direct piezoelectric effect), and conversely, when an electrical charge is applied, strains/stresses can appear in bone (converse piezoelectric effect) (Fernández et al., 2012a; Fernández et al., 2012c) (see Figure 1B). Similar to other studies (Fotiadis et al., 1999; Qin and Ye, 2004; Fernández et al., 2012a, Fernández et al., 2012c; Duarte et al., 2018), the bone was assumed to behave like a crystal with hexagonal symmetry, i.e., the third-order piezoelectric stress tensor **ℰ** is defined by four values and the electric permittivity tensor (dielectric tensor) β is a diagonal matrix with two constants. These tensors can be written in the following matrix form:

$$\;ℰ\;=\left(\begin{array}{ccc} 0 & 0 & 0\\ 0 & 0 & 0\\ e_{31} & e_{31} & e_{33} \end{array}\;\;\;\;\begin{array}{ccc} e_{14} & e_{15} & 0\\ e_{15} & -e_{14} & 0\\ 0 & 0 & 0 \end{array}\right)\;\text{and}\;\beta =\left(\begin{array}{ccc} \beta_{11} & 0 & 0\\ 0 & \beta_{11} & 0\\ 0 & 0 & \beta_{33} \end{array}\right),$$ (11)

where the third principal direction represents the longitudinal direction of the tibia bone (Fernández et al., 2012c). Forces resulting from the musculoskeletal system (e.g., muscle forces) were applied as Neumann boundary conditions to the medial and lateral condyles. Further, the following coupled linear variational equations were solved to calculate the displacement field u and the electric potential φ.

$$\int_{\Omega }2\mu \left(\rho \right)\varepsilon \left(u\right):\varepsilon \left(v\right)+\;\lambda \left(\rho \right)\text{Tr}\left(\varepsilon \left(u\right)\right)\text{Tr}\left(\varepsilon \left(v\right)\right)dx=\int_{\Omega }f_{B}\left(t\right)\cdot vdx$$

$$\;+\;\int_{\Gamma_{N}}f_{N}\left(t\right)\cdot vd\Gamma -\;\int_{\Omega }\left(\rho {\left(t\right)}^{\gamma }{ℰ}^{*}\nabla \varphi \left(t\right),\varepsilon \left(v\right)\;\right)dx,$$ (12)

$$\int_{\Omega }\left(\rho {\left(t\right)}^{\gamma }\;\beta \;\nabla \varphi \left(t\right),\;\nabla \psi \right)dx=\int_{\Omega }q_{B}\left(t\right)\;\psi \;dx$$

$$\;+\;\int_{{\text{Γ}}_{N}}q_{N}\left(t\right)\;\psi \;d\Gamma +\;\int_{\Omega }\left(\rho {\left(t\right)}^{\gamma }ℰ\left(u\left(t\right)\right),\nabla \psi \right)dx,$$ (13)

where Tr denotes the classical trace operator, v and ψ  are the test functions, dx denotes the differential element for integration over domain Ω, and $q_{B}=\text{div }D$. For simplicity, inertial effects were neglected and the bone remodeling formulation was restricted to isothermal and quasi-static conditions. More details about this model can be found in (Fernández, 2010; Fernández et al., 2010; Fernández et al., 2012a).

**TABLE 1:** Values of the parameters used in the finite element analyses.

Parameter	Name	Quantity	Reference
*ρ*_*a*_	Minimum bone density	0.010 g/cm^3^	Weinans et al. (1992); Fernández et al. (2010)
*ρ*_*b*_	Maximum bone density	1.740 g/cm^3^	
*S*_*r*_	Reference stimulus	0.004 J/g	
*B*	Experimental constant	1 (gcm^−3^)^2^ (MPa day)^−1^	
γ	Constitutive constant	3	
*M*	Constitutive constant	3,790 MPa/(cm^3^/g)^2^		Weinans et al. (1992), Bouguecha et al. (2011)
$f_{B}$	Body force	0 N/m^2^		Fernández et al. (2012a)
**Piezoelectric coefficients**			
$e_{31}$		1.507 × 10^−9^ C/mm^2^	Fotiadis et al. (1999), Fernández et al. (2012a)
$e_{33}$	Piezoelectric coefficient	1.872 × 10^−9^ C/mm^2^	
$e_{15}$		3.576 × 10^−9^ C/mm^2^	
**Electric permittivity coefficients**			
β_*11*_	Permittivity coefficient	88.54 × 10^−12^ F/mm	Fotiadis et al. (1999), Fernández et al. (2012a)
β_33_		106.248 × 10^−12^ F/mm	

For the numerical implementation of this formulation in the Python-based open-source finite element software FEniCS (www.fenicsproject.org, version 2019.1.0, GNU-GPL) (Logg et al., 2012; Alnæs et al., 2015), a mixed-function space (vector function space for the mechanical displacement and scalar function space for the electric potential) was used with Lagrange elements of order 2. Throughout the study, the time derivatives were discretized using the explicit fourth-order Runge-Kutta method.

$$\rho_{n+1}=\rho_{n}+\frac{\Delta t}{6}\;\left(k_{1}\;+2k_{2}\;+2k_{3}\;+\;k_{4}\right),$$ (14)

$$k_{1}\;=f\left(t_{n},\;\rho_{n}\right),$$ (15)

$$k_{2}\;=f\left(t_{n}+\frac{\Delta t}{2},\;\rho_{n}+\Delta t\frac{k_{1}}{2}\right),$$ (16)

$$k_{3}\;=f\left(t_{n}+\frac{\Delta t}{2},\;\rho_{n}+\Delta t\frac{k_{2}}{2}\right),$$ (17)

$$k_{4}\;=f\left(t_{n}+\frac{\Delta t}{2},\;\rho_{n}+hk_{3}\right),$$ (18)

where Δ*t* is the size of the time increment, $\rho_{n+1}$ and $\rho_{n}$ represent the bone density for the new and the current time step, respectively, and $f\left(t_{n},\rho_{n}\right)=B\left(\frac{U\left(\sigma \left(u\right),\varepsilon \left(u\right)\right)}{\rho_{n}}-S_{r}\right)$. The values of the parameters used in the following simulations are listed in Table 1.

### Setting up a Finite Element Model Using the Open-Source Framework

**FIGURE 2:**
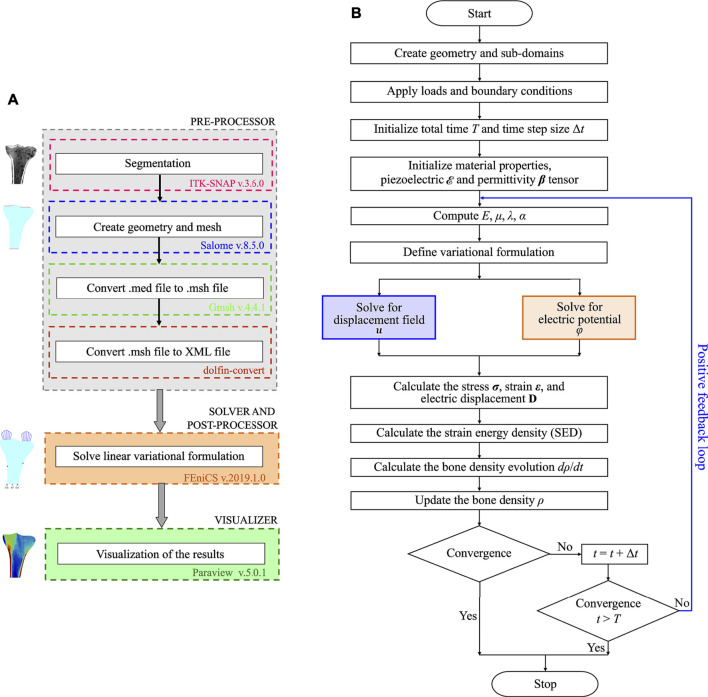
**(A)** Open-source software framework used to simulate tibial bone remodeling. **(B)** Flowchart of the piezoelectric bone remodeling algorithm implemented in the open-source finite element software FEniCS.

In this study, open-source software were used to ensure reproducibility of the simulation results, which is important for the evaluation of scientific work. Within our implementation of the open-source framework (Figure 2A), the Python-based open-source software packages used were ITK-SNAP (http://www.itksnap.org/, version 3.6.0, GNU-GPL) (Yushkevich et al., 2006) for segmentation, Salome (https://www.salome-platform.org/, version 8.5.0, GNU-LGPL) (Ribes and Caremoli, 2007) for designing and meshing, Gmsh (http://gmsh.info/, version 4.4.1, GNU-GPL) (Geuzaine and Remacle, 2009) and command-line tool dolfin-convert (GNU-LGPL) for pre-processing, the finite element software FEniCS for solving and post-processing, and Paraview (https://www.paraview.org/, version 5.0.1, 3-Clause BSD License) (Ahrens et al., 2005) for visualizing the simulation results. More details of the steps followed to set up the finite element model are published in (Bansod et al., 2021; Bansod and van Rienen, 2019). Figure 2B outlines the implemented bone remodeling algorithm, where the material properties were updated in each iteration through the positive feedback loop. The piezoelectricity of bone was incorporated into the algorithm by considering the matrix piezoelectricity (see Eq. 11) (Fernández et al., 2012a; Mohammadkhah et al., 2019). Consequently, the bone density distribution predicted by the finite element model is for dry bone. Following Open Science principles, the source code developed in this study is available at https://github.com/YDBansod/Bone_Remodelling.

### Loads and Boundary Conditions

**FIGURE 3:**
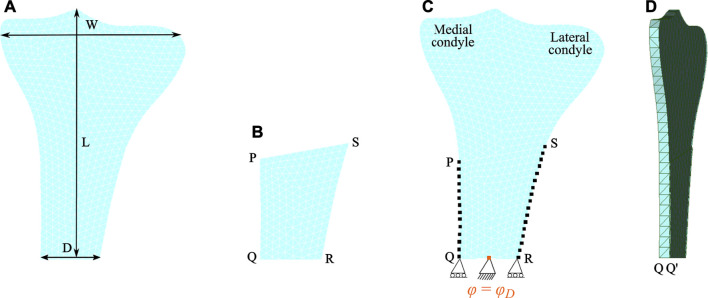
Finite element meshed models: **(A)** the human proximal tibia and **(B)** the side-plate. **(C)** Both tibia and side-plate were connected to each other at medial nodes (on the edge P-Q) and lateral nodes (on the edge S-R) highlighted by black dots. Note that the electrical boundary conditions are highlighted in orange. **(D)** Lateral view showing the thickness of the proximal tibia.

This study focuses on tibial bone remodeling under electrical stimulation, which has not yet been investigated. The tibia geometry was meshed using 10-node isoparametric tetrahedral elements. The mesh includes 4,132 elements and 1,507 nodes (see Figure 3A). The end-to-end distance between the medial and lateral condyles *W* is 71.10 mm and the diameter of the diaphysis *D* is 21.90 mm. The height of the proximal tibia *L* is 108.68 mm. Here, the lateral condyle-plateau is approximately 1.68 times that of the medial condyle-plateau. An obvious drawback of this two-dimensional model is the lack of connection between the cortical diaphysis regions. As a solution to this problem, an additional side-plate was considered (Figure 3B) joining these regions similar to that of the two-dimensional femur (Weinans et al., 1992; Fischer et al., 1997; Fernández et al., 2010; Garijo et al., 2014; Bansod et al., 2021). The cortical regions were joined only at medial and lateral nodes highlighted with black dots (see Figure 3C). The side-plate P-Q-R-S consists of 1,439 and 562 elements and nodes, respectively, and had mechanical properties analogous to the cortical bone. Additionally, the remodeling capacities of this plate were restricted.

Two Dirichlet boundary conditions were imposed at the lower region of the tibia, where the bottom face was restrained in the vertical direction, and to prevent rigid body movement, it was centrally constrained in both vertical and horizontal directions (see Figure 3C) (Nyman et al., 2004). Additionally, this central edge was electrically grounded (electric potential = 0 V). To perform multiplication of tensors of different orders, the tibia and the side-plate were modelled as a slice with a uniform thickness of 1 and 0.1 mm, respectively. Since partial differential equation (PDE) solver FEniCS does not support hexahedral mesh, both domains were meshed using tetrahedral elements with one element along the thickness (see Figure 3D). The anterior and posterior faces of the tibia and the side-plate were constrained along the *z*-axis to impose plane strain conditions. The total time of simulation and step size were set as *t* = 300 days (i.e., remodeling period) and Δ*t* = 0.125 days, respectively.

**FIGURE 4:**
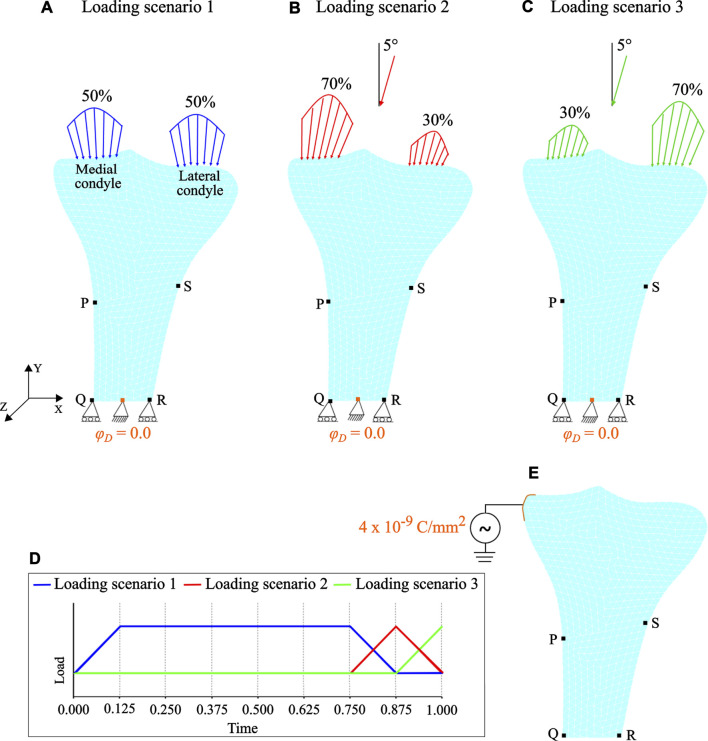
Loading and boundary conditions: **(A)** loading scenario 1 **(B)** loading scenario 2, and **(C)** loading scenario 3. Mark that for loading scenarios 2 and 3, the joint reaction force is inclined by 5^∘^ to the vertical direction. **(D)** Loading pattern applied independently and sequentially. **(E)** Therapeutic electrical stimulation in the form of surface electric charge applied to the lower end of the medial condyle during the period of reduced physical activity. Note that the electrical boundary conditions are highlighted in orange.

Forces resulting from muscle activities were applied as Neumann boundary conditions to the medial and lateral condyles. Mechanical loading conditions corresponding to the walking activity were simulated through the joint reaction force (approximately 3 times bodyweight of 70 kg) at the condylar region (Duda et al., 2001; Nyman et al., 2004; Perez et al., 2010; Quilez et al., 2017). The walking activity was considered to be represented by three cyclic loading scenarios. In the first loading scenario, the joint reaction force was distributed equally to each condyle in a vertical direction (Figure 4A). In the second loading scenario, the joint reaction force was distributed 70% over the medial condyle and 30% over the lateral condyle, while in the third loading scenario it was distributed 30 and 70% over the medial and lateral condyles, respectively. In the second and third loading scenarios, the joint reaction force was inclined 5^∘^ to the vertical direction such that its horizontal-component was directed towards the medial region (see Figures 4B,C) (Nyman et al., 2004; Perez et al., 2010; Quilez et al., 2017). Each loading scenario consists of a pair of parabolic distributed loads acting over the condyles and the load values considered are presented in Table 2. These cyclic loading scenarios with different frequencies were applied independently and consecutively (Figure 4D), where each iteration represents a day. The simulation was initially run for *t* = 300 days and with the obtained density distribution as an initial configuration, it was further assumed that between 300 and 400 days, the daily physical activity of the person was reduced due to injury or post-surgery. To incorporate this, the mechanical loads were applied once every 4 days between days 300–400 and referred to as “reduced physical activity” by Fernández et al. (2012a). Similar to piezoelectric material, when electrical stimulation is applied to bone, a corresponding mechanical displacement is obtained, which in this case leads to a change in bone density. Accordingly, during the reduced physical activity period (i.e., days 300–400), a surface electric charge *q*_*N*_ = 4 × 10^−9^ C/mm^2^ was applied to the lower end of the medial condyle (see Figure 4E).

**TABLE 2:** Values of the forces applied on each condyle (Perez et al., 2010; Quilez et al., 2017).

Loading scenario	Cycles/day	Loads applied at the medial condyle (N)		Loads applied at the lateral condyle (N)	
		*X*-axis	*Y*-axis	*X*-axis	*Y*-axis
1	3,000	0.0	−1,062.08	0.0	−1,062.08
2	500	−129.6	−1,353.28	−55.68	−634.88
3	500	55.68	−634.88	129.6	−1,353.28

The bone remodeling simulations started with a uniform initial density and by imposing the relevant boundary conditions (mechanical and electrical), the evolution of bone density was calculated. Here, the uniform initial bone density $\rho_{0}$ of 0.8 g/cm^3^ was selected because it is the mean of the bone density values (minimum 0.001 g/cm^3^ and maximum 1.74 g/cm^3^) used in the simulations (see Table 1). Starting from homogeneous density distribution, bone changes its shape in response to the applied external loads and at the end of the simulation, shows heterogeneous bone density distribution. In the present study, the bone density evolution was computed using the element-based approach (i.e., here the density was assumed to be constant elementwise). Further, the bone was considered to be homogeneous and isotropic linear-elastic material. Small displacement theory was assumed throughout this study (i.e., the displacement is so small that the deformed configuration of bone is not much different from the original shape). A direct LU solver was used to solve the linear asymmetric system. The simulations were performed on a computer with an Intel(R) Xeon(R) CPU E5-2687 W v6 @ 3.10GHz, 256GB RAM, and eight physical cores using FEniCS and it took approximately 12 s for each time-iteration.

### Parametric Study

Since the implemented bone remodeling algorithm is complex, iterative, and includes several parameters, a detailed parametric study is necessary. The influence of model parameters such as the uniform initial bone density $\rho_{0}$ and the reference stimulus *S*_*r*_ on the final bone density distribution was investigated. Concerning the initial bone density, five different values within the range of 0.2–1.4 g/cm^3^ and for the reference stimulus, six values within the range of 0.002–0.008 J/g were considered. Additionally, the variations within a range of ±1%, ±3%, and ±5% of the selected initial bone density of 0.8 g/cm^3^ were also considered to evaluate the model performance. The other model parameters were kept unchanged while performing these studies. The time step and mesh convergence studies were also conducted, and optimum mesh size of 1 mm and time step of 0.125 days were used in all simulations.

### Conversion of Hounsfield Units Into Bone Mineral Density

This study is based on a fresh human tibia specimen from a female donor (58 years old and 163 cm height) without any history of orthopedic injury or surgery. The specimen was scanned using CT (Aquilion 32, Toshiba, Neuss, Germany) and the images were saved in the digital imaging and communications in medicine (DICOM) format. By importing these images into AMIRA^®^ software (v.5.4.1, Zuse Institute Berlin, Berlin, Germany; Thermo Fisher Scientific, Waltham, MA, United States), the bone surface was segmented. In the literature, there are several relations relating HU to apparent bone density (Wirtz et al., 2000; Peng et al., 2007; Knowles et al., 2016). In the present study, to associate the HU values with the bone density at every point *i* of the tibia bone, the equation from Garijo et al. (2017) was modified as follows:

$$\rho_{i}=\rho_{a}+\;\frac{\rho_{b}-\rho_{a}}{HU_{max}-\;HU_{min}}\;\left(HU_{i}-\;HU_{min}\right).$$ (19)

where the HU maximum ($HU_{max}$) and minimum ($HU_{min}$) values were obtained from the CT data and correlated with bone density values of $\rho_{b}$ = 1.74 g/cm^3^ (cortical bone) and $\rho_{a}$ = 0.01 g/cm^3^ (resorbed bone), respectively (Weinans et al., 1992; Fernández et al., 2012a). The study was approved by the local ethical committee (registration number A2017-0110). Concerning the validation of simulation results, the predicted bone density distributions were qualitatively and quantitatively compared with the values obtained from the CT data. More precisely, for qualitative analysis, the obtained density distribution was visually matched with the CT image of the same tibia. Additionally, for quantitative analysis, the root mean square (RMS) error and mean deviation (MD) were calculated using the absolute differences between the bone densities estimated from the simulation and those from the CT. The nodal coordinates obtained from the finite element model were used to find the identical locations on the referent CT to calculate the corresponding bone mineral density (BMD) values.

## Results and Discussion

### Piezoelectric Bone Remodeling Simulation

**FIGURE 5:**
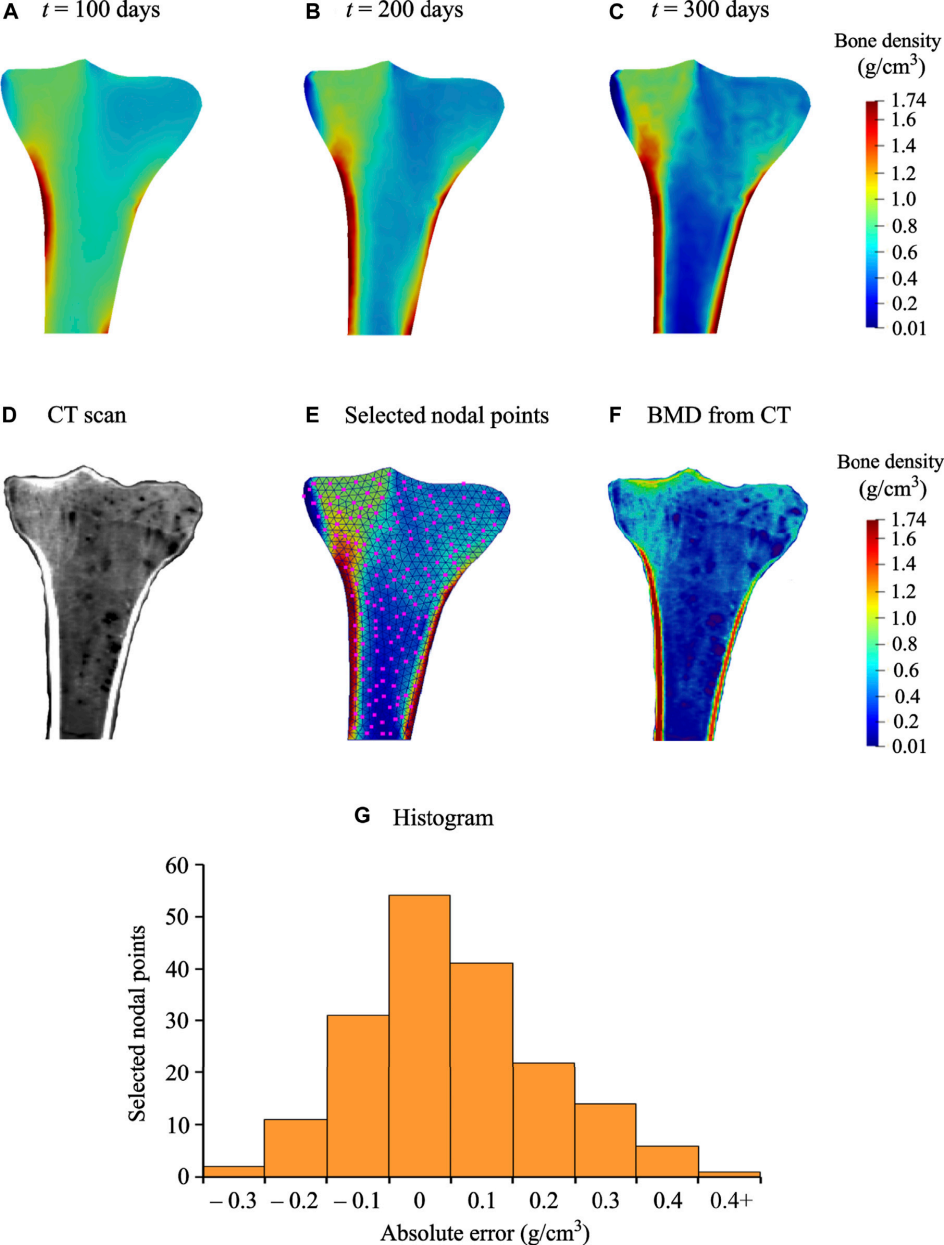
Evolution of bone density at time: **(A)** *t* = 100 days, **(B)** *t* = 200 days, and **(C)** *t* = 300 days. **(D)** Qualitative comparison of predicted bone density with the CT scan of the proximal tibia. **(E)** Location of the manually selected 182 nodal points for computing the RMS error and MD. **(F)** Quantitative comparison of predicted bone density with the BMD calculated from the CT **(G)** Histogram of absolute error (g/cm^3^) for the selected nodal points.

Starting from uniform density distribution, Figures 5A–C shows the evolution of the bone density at the intermediate and final time instants calculated using an interpolation post-processing technique. Regarding qualitative analysis, the final density distribution (see Figure 5C) was visually compared to the CT scan of the human proximal tibia (Figure 5D). The end configuration predicted fairly accurate density distribution with the trabecular bone beneath the tibial plateau, greater bone density in the medial region than in the lateral region, intramedullary canal with little trabecular bone, and cortical layers in the distal tibia. These observations are in good accordance with previous studies on tibial bone remodeling performed using commercial software (Rakotomanana, 2000; Perez et al., 2010; Robalo, 2011; Fang et al., 2013; Quilez et al., 2017). This provides a preliminary validation of the simulations performed with the open-source software framework illustrated in Figure 2A.

For quantitative analysis, RMS error and MD were computed for 182 nodal points (Figure 5E) that were selected using a non-probability convenience sampling technique (Lombardo et al., 2017). The HU values (−548 to 1883 HU) resulting from CT scans are in good accordance with those reported by Perez et al. (2007), Perez et al. (2010). Cortical bone was mostly observed in the cortex region, while trabecular bone was seen in the proximal tibia and only little in the medullary canal. The BMD calculated using the HU-density relationship described in *Conversion of Hounsfield units into bone mineral density* is shown in Figure 4F. By comparing the predicted (Figure 5C) and CT bone densities (Figure 5F) only for tibial domain (i.e., no side-plate nodes were considered), the RMS and MD values were computed to be 0.173 g/cm^3^ and 0.081 g/cm^3^, respectively. These quantitative comparisons provide supplementary validation of the implemented bone remodeling algorithm in open-source software. Further, for the selected nodal points, the histogram of the absolute errors is plotted in Figure 5G. In order to achieve better correspondence between simulation results and CT data, patient-specific distribution of uniform initial bone density, three-dimensional model with realistic boundary conditions, or a mixture of these should be considered. The findings presented here are intended to be representative of the tibial remodeling in general. Therefore, the approach used in this study is competent to predict the bone density distribution in 2D proximal tibia reasonably well.

### Influence of the Uniform Initial Bone Density

**FIGURE 6:**
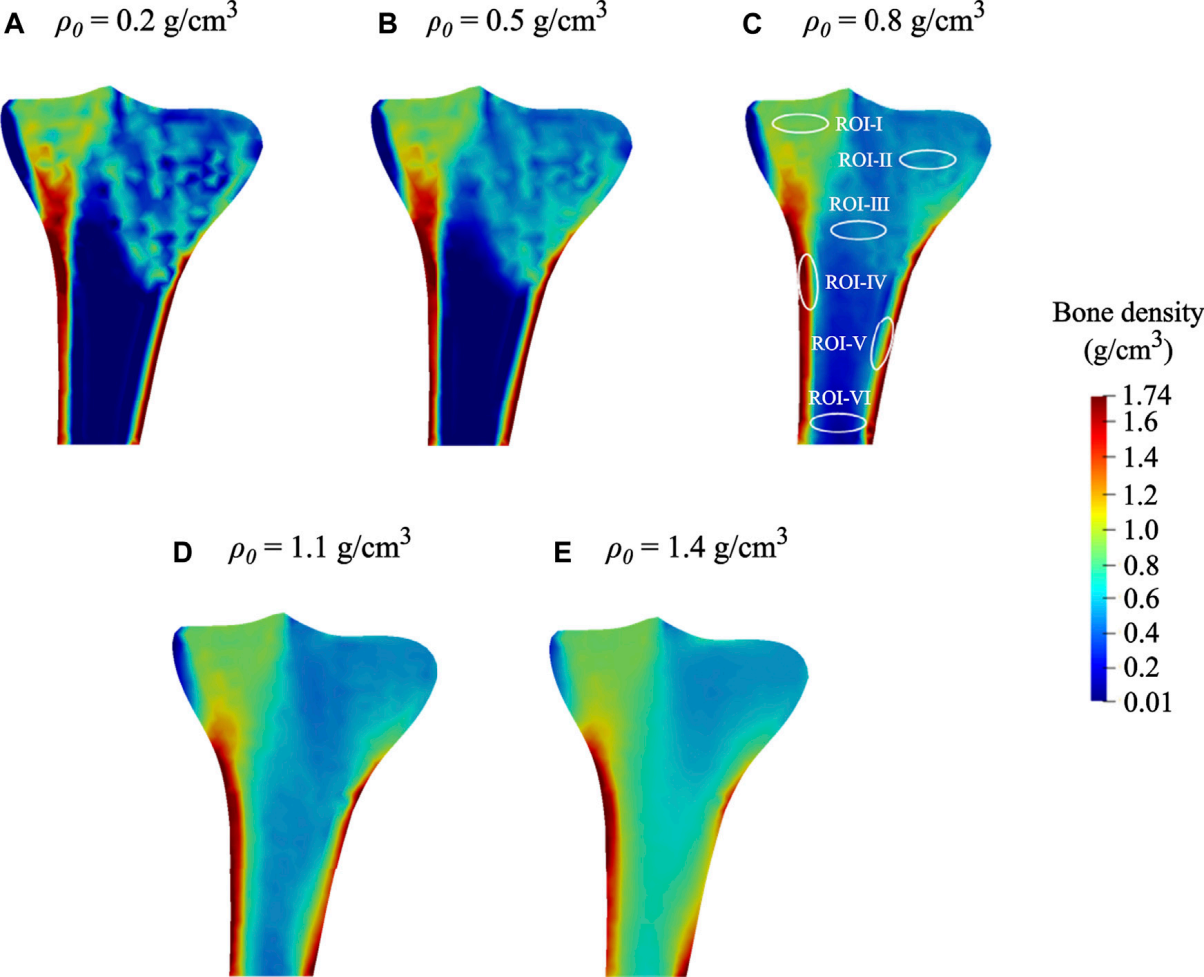
Final bone density distributions (*t* = 300 days) obtained for distinct values of uniform initial density: **(A)** *ρ*_0_= 0.2 g/cm^3^. **(B)** *ρ*_0_= 0.5 g/cm^3^, **(C)** *ρ*_0_= 0.8 g/cm^3^ (local regions of interests (ROIs) highlighted with white ellipses), **(D)** *ρ*_0_= 1.1 g/cm^3^, and **(E)** *ρ*_0_= 1.4 g/cm^3^.

Many bone remodeling models using different values of uniform initial bone density yield comparable but not identical final bone density distribution. In order to investigate the effect of varying initial bone density $\rho_{0}$ on the final density distribution, the model was analyzed for five different values of 0.2 g/cm^3^, 0.5 g/cm^3^, 0.8 g/cm^3^, 1.1 g/cm^3^, 1.4 g/cm^3^ and the obtained results are shown in Figure 6. As seen here, the final density distributions are not entirely identical and extensively rely on the value of uniform initial density. These findings are analogous to previous studies (Weinans et al., 1989; Orr, 1992; Turner et al., 1993) that also used the element-based remodeling algorithm.

**FIGURE 7:**
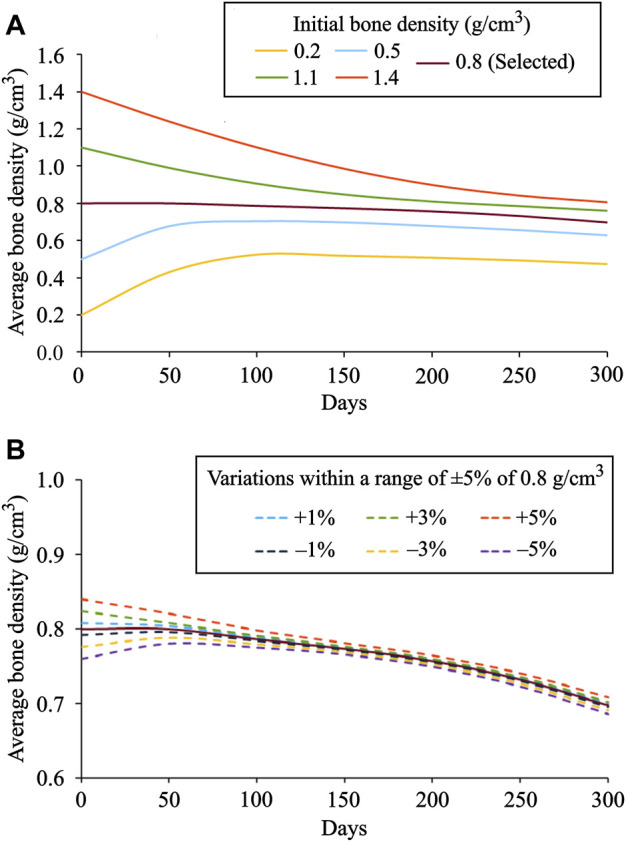
Evolution of average bone density for: **(A)** distinct values of initial density ranging from 0.2 to 1.4 g/cm^3^ and **(B)** small variations within a range of ±1%, ±3%, and ±5% introduced to the selected initial bone density of 0.8 g/cm^3^.

At time 0 days, for the initial bone density equal to or higher than 0.8 g/cm^3^, the average bone density gradually declined throughout the remodeling period (see Figure 7A). On the contrary, when the same was equal to or lower than 0.5 g/cm^3^, the average bone density increased steadily in the first 75 days and then flattened out. Starting from different initial bone density, the resulting average bone densities tend to converge as time advances. On the 0th, 150th, and 300th day, the average bone density ranged from 0.2 to 1.4 g/cm^3^, 0.51–0.98 g/cm^3^, and 0.47–0.90 g/cm^3^, respectively. Further, the relative difference between these density values on the 300th day lies between 5.7 and 20.9%. Moreover, the bone density distributions obtained for different initial density values are also dissimilar (see Figure 6). In this figure, the region with the highest bone density (red) was reduced with an increase in initial density resulting in lower average bone density and this is in sync with the tendency noticed in Figure 7A.

As seen in Figure 7B, when variations within a range of ±1%, ±3%, and ±5% were introduced into the selected initial bone density of 0.8 g/cm^3^, the obtained density distribution at the final time was similar but not identical. It was thus observed that the bone density distribution (Figure 6) and the average bone density (Figures 7A,B) are dependent on the chosen value of initial density. However, the final density distribution shouldn’t be dependent on the initial conditions because they are mere numerical assumptions and not supported by substantial evidence. Therefore, to reduce such dependency, the lazy or dead zone (Carter, 1984; Huiskes et al., 1987) together with the saturated density change rate (Martínez-Reina et al., 2016; Su et al., 2019) could be incorporated into the remodeling algorithm implemented here.

**FIGURE 8:**
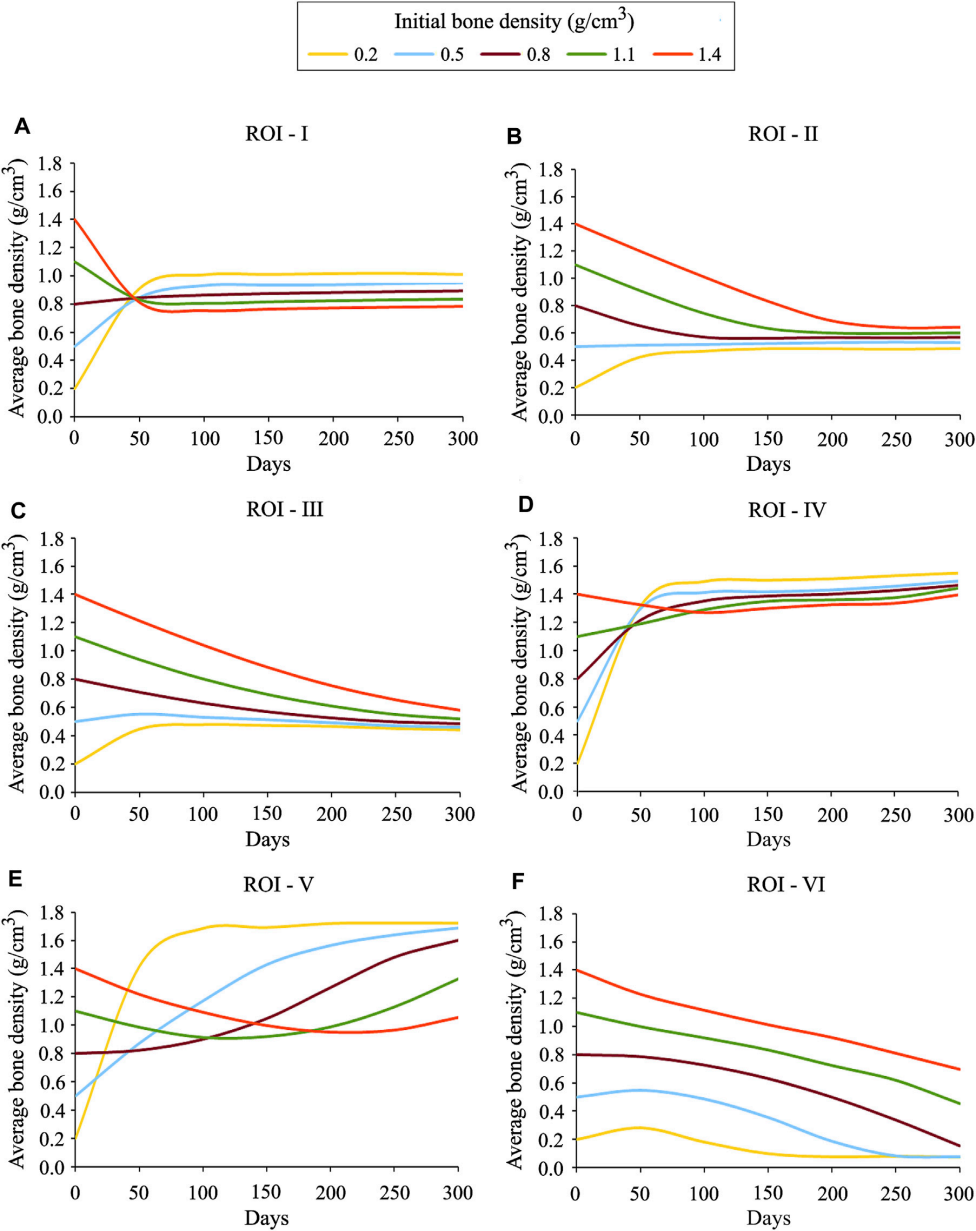
Average bone density evolution in the local regions of interest marked in Figure 6C: **(A)** ROI I **(B)** ROI II **(C)** ROI III **(D)** ROI IV **(E)** ROI V and **(F)** ROI VI.

In order to investigate the local bone adaptation, the evolution of average bone density in six regions of interest (ROIs) highlighted with white ellipses (see Figure 6C) was plotted in Figure 8. With the reference to the initial bone density of 0.8 g/cm^3^ in ROIs I, IV, and V, the average bone density increased for the lower values, whereas for the higher ones, it decreased over the remodeling period (see Figures 8A,D,E). Regarding ROIs I-IV, as days proceed, the differences in the average bone density were getting smaller until they were negligible at the final time (see Figures 8A–D). Also, a tendency to converge to a common value is remarkable. In the case of ROIs V and VI, the resulting average bone density deviated from each other over the bone remodeling span (see Figure 8 E and F). Therefore, this study shows that the final bone density distribution is location-specific. Here, the simulation results are based on the uniform initial bone density assumption opposing to the reality, but it would be meaningful for the study of numerical algorithms. Therefore, for interpreting the results, it is more logical to focus on the final bone density distribution instead of the evolution of density over time.

### Influence of the Reference Stimulus

**FIGURE 9:**
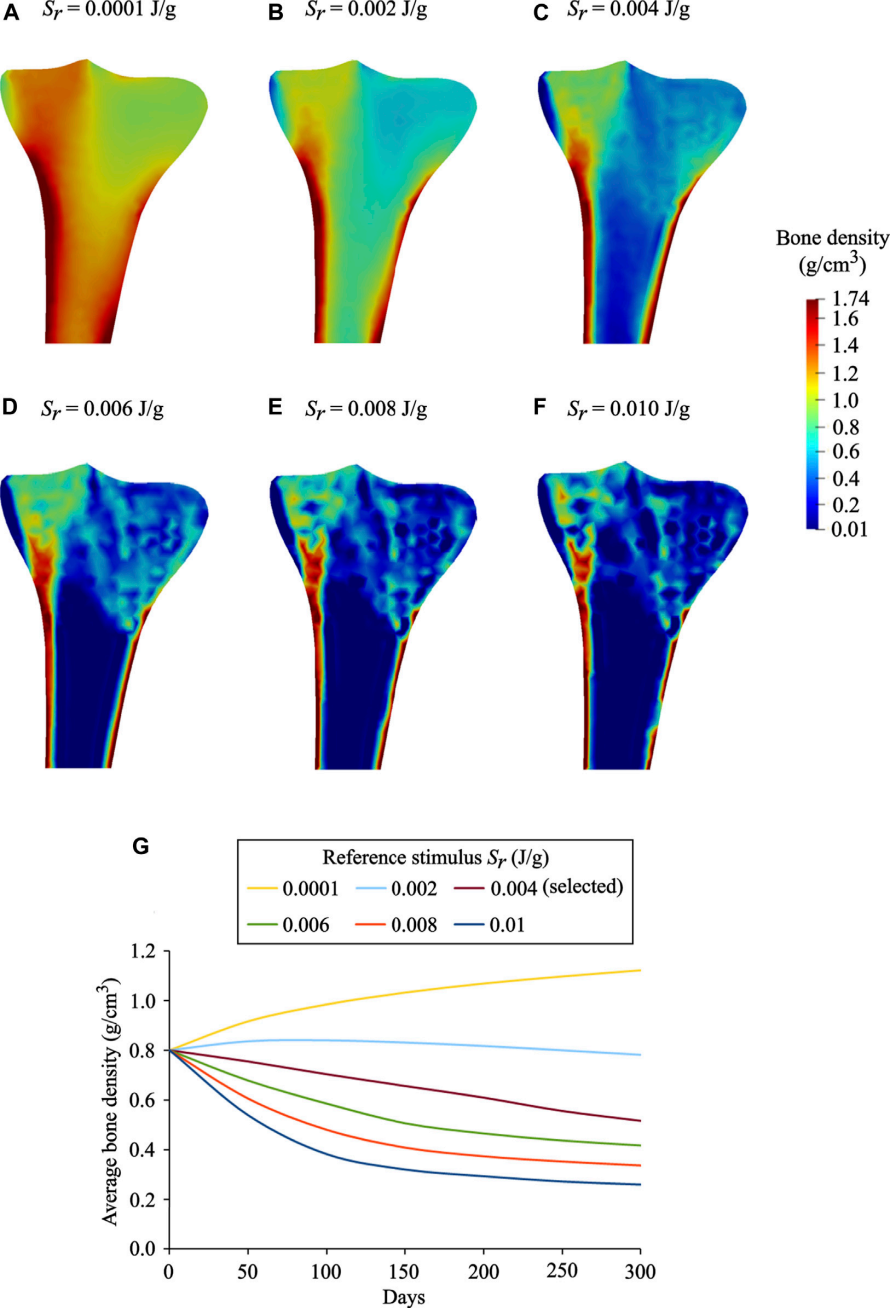
Final bone density distributions (*t* = 300 days) obtained for distinct values of reference stimulus: **(A)** *S*_*r*_ = 0.0001 J/g, **(B)** *S*_*r*_ = 0.002 J/g **(C)** *S*_*r*_ = 0.004 J/g (selected), **(D)** *S*_*r*_ = 0.006 J/g **(E)** *S*_*r*_ = 0.008 J/g, and **(F)** *S*_*r*_ = 0.010 J/g. **(G)** Evolution of average bone density obtained for the reference stimulus values ranging from 0.0001 J/g to 0.01 J/g.

The reference stimulus *S*_*r*_ has a considerable effect on bone remodeling simulations. The results obtained for *S*_*r*_ = 0.0001 J/g, 0.002 J/g, 0.004 J/g, 0.006 J/g, 0.008 J/g, and 0.01 J/g are depicted in Figure 9 A–F. It was observed that with an increase in the reference stimulus value, the highest bone density region (red) was reduced, and concomitantly the lowest bone density region (blue) was enlarged. Similar observations have been reported by Sarikanat and Yildiz (2011). In the present study, the reference stimulus of 0.004 J/g was selected as it provides the density distribution, which is in good agreement with the real bone. For the reference stimulus values less than 0.004 J/g, the average bone densities increased steadily, while for greater values, the average bone densities decreased steadily at a similar rate (Figure 9G). On the 300th day, for the reference stimulus values from 0.0001 J/g to 0.01 J/g, the resulting average bone density ranges from 1.12 g/cm^3^ to 0.28 g/cm^3^. It is remarkable that a lower value of the reference stimulus resulted in higher average bone density. Therefore, the bone density distribution and the average bone density (Figure 9) are greatly dependent on the reference stimulus value. However, in reality, bone tissue is not very sensitive to changes in the reference mechanical stimulus. Taking this into account, many studies have considered a nonlinear remodeling rate relation including the lazy or dead zone (Carter, 1984; Huiskes et al., 1987; Fischer et al., 1995).

### Bone Piezoelectricity

**FIGURE 10:**
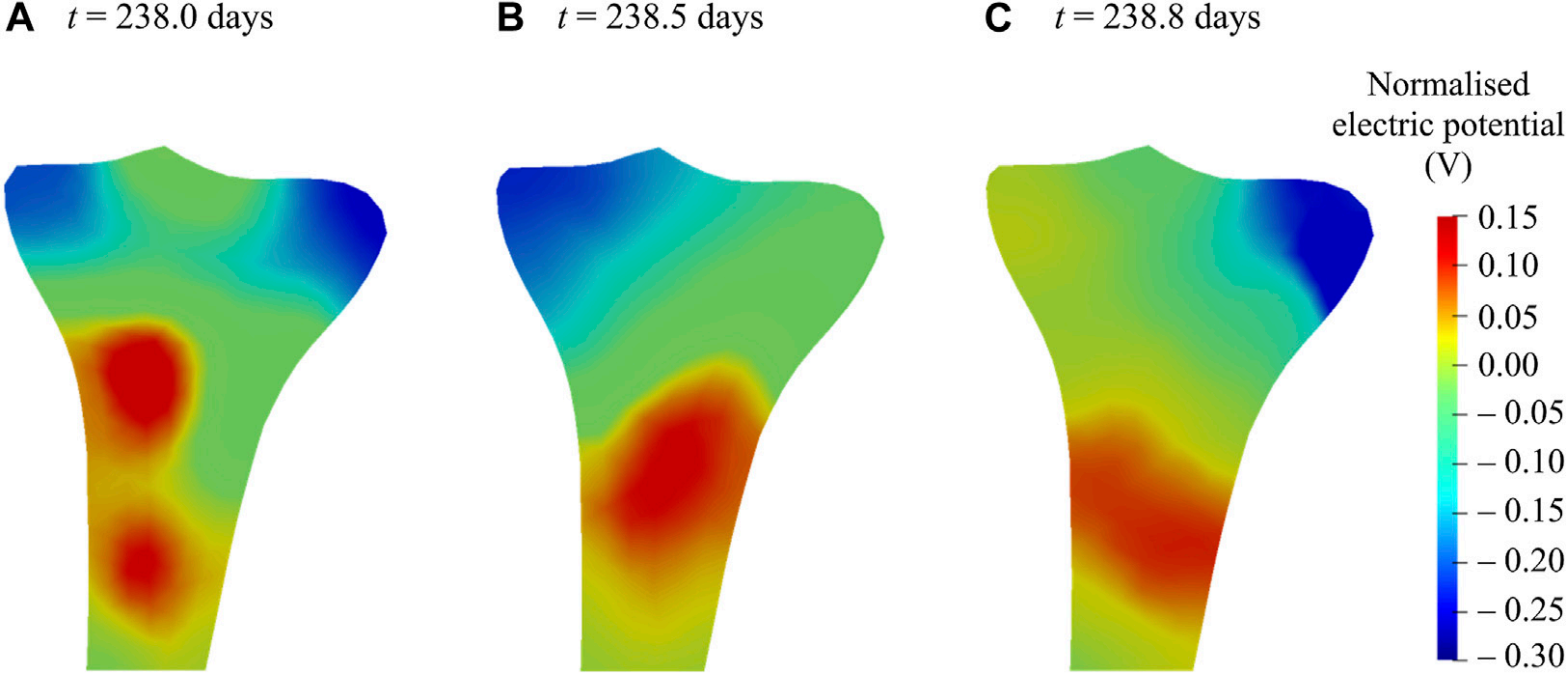
Normalized electric potentials generated at various time instants of the 238th day due to the application of varying mechanical loads during walking.

It is evident from experimental studies (Fukada and Yasuda, 1957; Bassett and Becker, 1962; Becker, 1964; Bassett et al., 1964; Black and Korostoff, 1974; Yasuda, 1977; Nade, 1994; Zigman et al., 2013) that the mechanical loading induces changes in the bone electric potentials in a way that regions exposed to compressive loads generated negative potentials, whereas those exposed to tensile loads generated positive potentials. Figure 10 demonstrates changes in the distribution and magnitude of the electric potentials generated at different time instants of the day due to the application of varying mechanical loads, representing the direct piezoelectric effect. For better comparison within the generated electrical potentials, they were normalized with respect to their mean amplitude. For electromechanical simulations, negative potentials are associated with osteoblast-induced bone formation, whereas positive potentials are associated with osteoclast-induced bone resorption (Qin and Ye, 2004; Fernández et al., 2012a; Cerrolaza et al., 2017). These electrical potentials play a vital role in the process of bone healing and remodeling (Fukada and Yasuda, 1957; Fukada and Yasuda, 1964; Marino and Becker, 1970; Becker and Spadaro, 1972; Gjelsvik, 1973; Ramtani, 2008; Zigman et al., 2013). A sensitivity analysis was conducted to investigate the influence of piezoelectric and permittivity tensors on the generated electric potentials. This analysis demonstrated that the model was very sensitive to the values of these parameters and similar findings have been observed by Fernández et al. (2012c).

### Therapeutic Electrical Stimulation to Reduce Bone Loss

**FIGURE 11:**
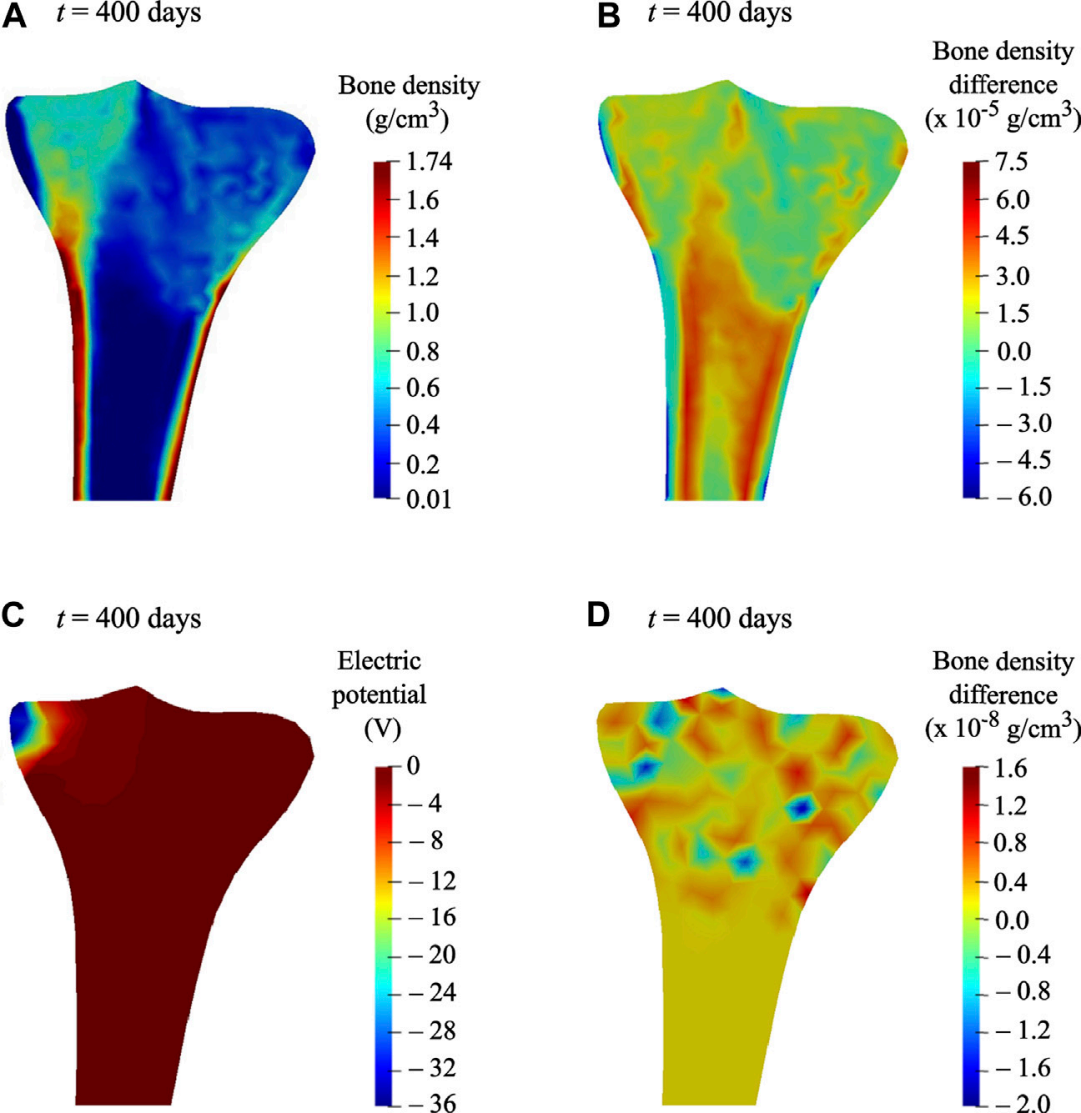
**(A)** Predicted bone density distribution after the reduced physical activity period (*t* = 400 days). **(B)** Differences in predicted bone density, after the remodeling period (*t* = 300 days) and after the reduced physical activity period (*t* = 400 days). **(C)** Electric potential generated at time *t* = 400 days, when a surface electric charge was applied to the medial condyle for the reduced physical activity period (i.e., 300–400 days). **(D)** Differences in predicted bone density at time *t* = 400 days, when therapeutic electrical stimulation was applied along with acting mechanical loads and when only mechanical loads were acting.

The bone density distribution predicted after the remodeling period (Figure 5C) was assumed to be an initial state for the simulations of reduced physical activity and therapeutic electrical stimulation (see *Loads and boundary conditions* for more details). The density distribution predicted after the reduced physical activity period is shown in Figure 11A. In order to investigate the local variations in bone density distributions as a result of the reduced physical activity, the differences between bone density after the remodeling period (i.e., on 300th day) and after the reduced physical activity period (i.e., on 400th day) are plotted in Figure 11B. It is notable that the density distribution in both condyles and the intramedullary canal was lower than that obtained after the remodeling period.

The most significant benefit of including the piezoelectricity in bone remodeling study is the ability to change bone density under electrical stimulation (Fernández et al., 2012a) through the converse piezoelectric effect. When a surface electric charge was applied to the tibia during the reduced physical activity period, a negative potential of nearly −36 V was noticed (see Figure 11C). In order to show the resulting change in bone density, differences between densities while the electric charge was applied in addition to the acting mechanical loads and while only the mechanical loads were acting, are plotted in Figure 11D. Minor variations in bone density were observed, where areas in red and blue correspond to growth and reduction in bone density, respectively. In comparison with the reduction in bone density in condyles and intramedullary canal owing to reduced physical activity (Figure 11B), the therapeutic electrical stimulation affects only the area in its vicinity, resulting in a clear increase in the density between the condyles (Figure 11D). These preliminary observations showed that the electromechanical loads affect the bone density evolution. Hence, therapeutic electrical stimulation could be treated as a supplement to improve bone remodeling and to minimize bone loss, e.g., physical impairment or osteoporosis. Direct comparison of the simulation predictions with the experimental data is not feasible because of its unavailability. Matrix piezoelectricity and streaming potential are the two mechanisms responsible for the piezoelectricity in bone (Fernández et al., 2012a; Cerrolaza et al., 2017; Mohammedkhah et al., 2019). However, in the present study, small changes in bone density after electrical stimulation were ascribed only to the matrix piezoelectricity. Therefore, the obtained results inspire further advancement of a multi-physics model that involves multiple coupled phenomena such as matrix piezoelectricity (Fukada and Yasuda, 1957), strain-generated fluid flow (Cowin et al., 1995), and streaming potential (Pollack et al., 1984) leads to more promising results for therapeutic electrical stimulation.

So far, only a small number of bone remodeling models have been studied considering the piezoelectric effect and that even using either commercial software or in-house programs that are not easily accessible. Nonetheless, in the current study, the piezoelectric strain-adaptive bone remodeling in the human proximal tibia was analyzed for the first time and that too using an open-source framework. The Python code files developed are available on GitHub for easy access. Here, the most contemporary model of bone remodeling that considers the piezoelectric properties of bone was implemented to investigate bone remodeling under electrical stimulation. The predicted bone density distributions were validated qualitatively by visually comparing with the radiographic scan and quantitatively by calculating the RMS error between the predicted BMD and the BMD obtained from the CT. Both direct and converse piezoelectric effects were shown by combining the effects of mechanical and electric fields on the response of bone. A detailed parametric study was carried out to investigate the influence of reference stimulus and uniform initial bone density on the final density distribution. For time discretization in bone remodeling studies, the explicit Euler method was commonly used, which is not very precise and for large time-step size, becomes unstable. Thus, in this study, a more stable and accurate fourth-order Runge-Kutta method was used. The findings of this study highlight the role of bone piezoelectricity in the therapeutic electrical stimulation and could be used clinically to improve the osseointegration of the electrically active implants.

Nonetheless, this study has some drawbacks. In spite of the substantial contribution of matrix piezoelectricity and streaming potential to the electromechanical properties of bone (Fernández et al., 2012c), their coupling is not yet well understood. In the literature, there are many discrepancies in the measured values of the piezoelectric strain tensors (Fukada, 1968; Fotiadis et al., 1999; Qin and Ye, 2004; Mohammadkhah et al., 2019). Here, for computational modeling purposes, the bone was assumed to be electrically homogeneous, which is not the case in reality (Johnson et al., 1980). This study does not take into account the external bone remodeling (Goda et al., 2016). The implemented algorithm does not consider the effect of gender, age, disease, and injury on the bone remodeling process. The findings of the present study cannot be generalized to the severely osteoporotic or fractured bone (i.e., with very less or no physical activity), as the piezoelectric bone remodeling model suggests replacing only the part of mechanical load resulting from the daily physical activity by electrical stimulation.

In future work, the constitutive laws for mechanical and electrical behavior of bone will be adapted to include coupled multi-physics phenomena such as matrix piezoelectricity and streaming potential to better predict bone density evolution. The simulations of reduced physical activity and electrical stimulation can be performed considering the initial bone density distribution to be patient-specific. The electric stimulation of varying magnitude will be applied to the different regions of the tibia investigating the effect of stimulation intensity and site of excitation on bone remodeling. To correlate HU values with BMD, a calibration phantom could be used instead of a calibration function. By including the results from musculoskeletal multibody simulations, the boundary conditions should be improved to perform full 3D simulations. From a rehabilitation perspective, future studies should investigate whether the numerical model is indicative of the potential therapeutic values of electrical stimulation. The implemented piezoelectric bone remodeling algorithm can also be employed for applications investigating the effect of electrically active implants (Schmidt et al., 2015; Zimmermann and van Rienen, 2015; Raben et al., 2019; Zimmermann et al., 2021) in the adjacent bone tissue with respect to peri-implant bone remodeling.

## Conclusion

This is the first attempt to investigate the piezoelectric bone remodeling in the human tibia for daily walking activity using an open-source framework. The simulation results predicted reasonably accurate bone density distributions that were validated both qualitatively and quantitatively against the CT data. The parametric analysis showed that different uniform initial bone density and reference stimulus values resulted in different density distribution at the final time. A reduction in bone density was observed for the reduced physical activity compared to the daily physical activity. Therapeutic electrical stimulation applied over a period of reduced physical activity showed increased bone deposition suggesting that in the case of bone loss, e.g., physical impairment or osteoporosis, the mechanical loads can be replaced in part by electrical stimuli that enhance bone density or reduce bone loss.

## Data Availability

The datasets presented in this study can be found in online repositories. The names of the repository/repositories and accession number(s) can be found below: https://github.com/YDBansod/Bone_Remodelling.
